# TRANSLATION AND VALIDATION OF THE BRAZILIAN VERSION OF THE “QUALITY OF LIFE ASSESSMENT IN SPINA BIFIDA” QUESTIONNAIRE FOR CHILDREN AND TEENAGERS

**DOI:** 10.1590/1984-0462/2021/39/2019312

**Published:** 2020-08-28

**Authors:** Jorge Pompermaier, Maria Cristina de Andrade, Marcela Leal da Cruz, Antonio Macedo

**Affiliations:** a Núcleo de Urologia Pediátrica, São Paulo, SP, Brazil.; b Universidade Federal de São Paulo, São Paulo, SP, Brazil.

**Keywords:** Bifida, spina, Quality of life, Children, Espinha bífida, Qualidade de vida, Criança

## Abstract

**Objective:**

To assess the reliability and validity of the Quality of Life Assessment in Spina Bifida (QUALAS), children and teenager’s versions (QUALAS C and T, respectively). This is the first self-applicable quality of life assessment tool for patients under 13 years of age, which also addresses the issue of urinary and fecal incontinence.

**Methods:**

Two urologists performed the translation of both QUALAS versions. A commission produced a consensus version (Version 2), which was applied as a pilot study to define Version 3. It was then backtranslated into English and compared with the original version for equivalence of concepts. Internal consistency with Cronbach’s alpha and the intraclass correlation coefficient (ICC) reproducibility was analyzed after two assessments with an interval from two to four weeks. Convergent and divergent validities between the QUALAS and a generic health-related quality of life questionnaire, the KIDSCREEN-27, were studied through Pearson’s correlation.

**Results:**

The reliability analysis showed good internal consistency for QUALAS-C (α=0.73) and QUALAS-T (α=0.79) and good reproducibility in both questionnaires (QUALAS-C - ICC=0.86; QUALAS-T - ICC=0.92). For QUALAS-C convergent validity, there was a low correlation between its items (r=0.35). In addition, a low correlation was also found in the divergent validity analysis, when compared to the KIDSCREEN-27 (r≤0.33). Convergent and divergent validities of the QUALAS-T questionnaire had similar results: r=0.46 and r≤0.49, respectively.

**Conclusions:**

After the adaptation and validation process, QUALAS-C and QUALAS-T questionnaires showed to be reliable and valid instruments for measuring the health-related quality of life of children and teenagers with spina bifida aged 8 years or older.

## RESUMO

**Objetivo:**

Avaliar a confiabilidade e a validade do questionário QUALAS (*Quality of Life Assessment in Spina Bifida*) nas versões para crianças e adolescentes (QUALAS-C e QUALAS-T, respectivamente). Este é o primeiro instrumento autoaplicável de avaliação da qualidade de vida para pacientes menores de 13 anos e que também aborda a questão das incontinências urinária e fecal.

**Métodos:**

Dois urologistas realizaram a tradução das duas versões do QUALAS. Uma comissão produziu uma versão de consenso (Versão 2), a qual foi aplicada no estudo piloto para definir a Versão 3. Esta foi retrotraduzida para o inglês e comparada à versão original para equivalência de conceitos. Para verificar a confiabilidade, analisou-se a consistência interna com o alfa de Cronbach e a reprodutibilidade com o coeficiente de correlação intraclasse (CCI) após duas aplicações do questionário em intervalo de duas a quatro semanas. As validades convergente e divergente foram estudadas por meio da correlação de Pearson entre o QUALAS e um questionário genérico de qualidade de vida relacionada à saúde, o KIDSCREEN-27.

**Resultados:**

A análise de confiabilidade revelou que ambos os questionários apresentaram boa consistência interna (QUALAS-C - α=0,73; QUALAS-T - α=0,79) e boa reprodutibilidade (QUALAS-C - CCI=0,86; QUALAS-T - CCI=0,92). Na análise da validade convergente do QUALAS-C, observou-se baixa correlação entre os itens (r=0,35). Além disso, a análise da validade divergente também demonstrou baixa correlação quando comparada ao KIDSCREEN-27 (r≤0,33). As validades convergente e divergente do questionário QUALAS-T tiveram resultados semelhantes: r=0,46 e r≤0,49, respectivamente.

**Conclusões:**

Após o processo de adaptação e validação, pode-se afirmar que os questionários QUALAS-C e QUALAS-T são instrumentos confiáveis e válidos para a mensuração da qualidade de vida relacionada à saúde de crianças e adolescentes com espinha bífida a partir dos 8 anos de idade.

## INTRODUCTION

Spina bifida is the most common congenital defect of the central nervous system (CNS). Brazil occupies the fourth place in the world’s incidence rank, with 11.39 cases per 10,000 live births, behind only Bulgaria, Venezuela, and Mexico.^1^ Recent data show that 735 births of spina bifida carriers were registered in 2016 among 2,857,800 live births in the same year.^2^

Clinical follow-up and continuous medical treatment are essential for patients with spina bifida, considering the repercussions of physical, psychological and social orders on the quality of life (QoL) of these patients. The World Health Organization (WHO) currently defines QoL as “the individual’s perception of his or her position in life, in the cultural context and in the value system that he or she lives and in relation to their goals, expectations, standards, and concerns”.^3^ Published studies regarding the QoL in patients with spina bifida used several instruments, most of which were not elaborated or do not include important aspects, such as the influence of urinary incontinence and changes in bowel habits on their QoL. In addition, they were not validated for the Portuguese language.^4^

One of the most specific questionnaires, which can be easily completed and is available for patients with spina bifida aged between eight and 17, is the Quality of Life Assessment in Spina Bifida (QUALAS), which has a version for children - QUALAS-C and one for teenagers - (QUALAS-T). It is the first self-applicable QoL tool for patients under 13 years of age that also addresses the issue of urinary and fecal incontinences, which are important aspects for this specific population. It has been originally validated and proposed in English^5^ ^,^ ^6^ and validated in other languages, such as Japanese.^7^

Our objective was to translate and validate both versions of the QUALAS Questionnaire (C and T) into Portuguese language.

## METHOD

Authorization to carry out this study was obtained from the main authors of the original papers, and the study was registered and approved by the Research Ethics Committee from Universidade Federal de São Paulo.

The QUALAS questionnaire, both in its version for children aged eight to 12 and in the other for adolescents aged between 13 and 17, includes 10 questions divided into two domains with five questions each.^5^ ^,^ ^6^ Each of the questions has five to six alternatives arranged on a Likert scale. The total score of each domain varies from 0 to 100, and the higher the score, the higher the patient’s QoL. In the first application of the QUALAS questionnaire, the KIDSCREEN - 27 questionnaire was also applied, which is a generic instrument for assessing children and adolescents’ QoL.^8^ Between two and four weeks after the first application, patients were called and Version 3 of the QUALAS questionnaire was again applied to evaluate their test-retest reliability.

The translation and cultural adaptation processes of the questionnaires were carried out following the guidelines their main author. Two independent urologists translated the questionnaires into Portuguese and created, therefore, two versions that were analyzed by a commission that produced a consensus version for each questionnaire. This consensus version was used in the pilot study, in which semi-structured interviews were made with five patients and their caregivers to adjust the used language. This version of the questionnaires was again translated into English through the back-translation process by a native English-speaking translator, who had no contact with the original version of the questionnaire. The version after adjustments in the pilot study was used in the main study.

The study population consisted of a sample of convenience represented by children and adolescents aged between eight and 17, with a corrected diagnosis of spina bifida. Patients were chosen during routine medical care between February 2017 and March 2018. After the delivery, reading, and signing of the Informed Consent Form by the parents or caregivers and the Informed Assent Form to children and adolescents, the questionnaires were delivered by the researcher. The questionnaire was applied during the medical interview, and the investigators asked the questions orally.

The evaluation of the psychometric properties of an instrument is part of its validation process. The pilot questionnaire study allowed to evaluate its face and content validity and, thus, to identify the changes that were required for a better understanding of the questionnaire and then to evaluate if the instrument was measuring what it was proposed to measure.

Not only the main study allowed to evaluate other criteria of validity, but it also allowed to evaluate its reliability. One of the forms used to evaluate the questionnaire reliability was by measuring its internal consistency through the Cronbach’s alpha coefficient calculation. Reproducibility (through the test-retest method) is another key component for assessing reliability. The intraclass correlation coefficient (ICC) was used for its representation.

The convergent and divergent validities evaluate the independence or redundancy concept of the questionnaire items. Pearson’s correlation coefficient was calculated between the domains of each of the scales (QUALAS-C and QUALAS-T) and with the domains of the KIDSCREEN-27 instrument.

The sociodemographic and clinical characteristics were described by the number and percentage for the categorical variables and as mean±standard deviation for the quantitative variables. Internal consistency was assessed using the Cronbach’s alpha coefficient, with values between 0.70 and 0.90 that indicate a good consistency without redundancy. The test-retest reliability was evaluated using the ICC (values ≥0.70 indicate acceptable reliability). Differences in the mean of domains and total score between the two applicants were assessed by Student’s *t*-test. The convergent and divergent validities were analyzed by calculating Pearson’s correlation coefficient between the domains of each of the scales (QUALAS-C and QUALAS-T) and also with the domains of the KIDSCREEN-27 instrument.

Statistical analysis was performed using STATA/SE 15.1 for Windows software (StataCorp, USA), adopting a 5% significance level, which means that p<0.05 were considered as statistically significant.

## RESULTS

The study population consisted of 81 individuals, of whom 40 answered the QUALAS-C and 41, the QUALAS-T. Regarding the sociodemographic aspects of these patients, 65% were female in the QUALAS-C group and 63.4% in the QUALAS-T group. There was a total of 42.5% of Caucasians in the QUALAS-C group and 61% in the QUALAS-T group. African-Brazilians accounted for 30% in the QUALAS-C group and 26.8% in the QUALAS-T group, and multiethnic in 27.5% in the QUALAS-C group and 12.2% in the QUALAS-T group. Ventriculoperitoneal shunt was present in 72.5% of the QUALAS-C group and 68.3% of the QUALAS-T group. Twenty-eight (70%) patients of the QUALAS-C group and 24 (58.5%) are in the QUALAS-T group were already registered and in follow-up in our outpatient clinic, whereas the other cases were new referrals. The evaluation of urological aspects showed that 75% of the participants used clean intermittent catheterization (CIC), 70% had urinary incontinence, whereas 12.5% had bladder augmentation and 7.5% catheterizable channel in QUALAS-C patients. QUALAS-T group had 63.4% using CIC, 51.2% with urinary incontinence, 19.5% with bladder augmentation, and 9.8% with a catheterizable channel.

The internal consistency of the questionnaires was evaluated through Cronbach’s alpha, which for the QUALAS-C presented a value of 0.73 in the Total score, 0.56 for the Self-esteem and independence domain, and 0.74 for the Bladder and bowel domain. The Cronbach’s alpha values found for the QUALAS-T were 0.79 for the Total score, 0.71 for the Family and independence domain, and 0.74 for the Bladder and bowel domain. According to the obtained values, the Total score and the Bladder and bowel domain presented good internal consistency. Only the Self-esteem and independence domain of the QUALAS-C presented a value below the desirable (between 0.70 and 0.90).

Table 1 presents the test-retest reliability assessed between patients with an interval of two and four weeks between evaluations. It was evaluated by the ICC for each domain and total score. According to the results, an excellent agreement between the two applications was observed in both questionnaires.

**Table 1:** Intraclass correlation coefficient and its respective 95% confidence interval for QUALAS-C and QUALAS-T questionnaires.

Domain	QUALAS-C	
	ICC	95%CI
Self-esteem and independence	0.79	0.67-0.91
Bladder and bowel	0.78	0.65-0.90
Total score	0.86	0.78-0.94
Domain	QUALAS-T	
	ICC	95%CI
Family and independence	0.87	0.80-0.95
Bladder and bowel	0.91	0.86-0.96
Total score	0.92	0.87-0.97

CCI: coeficiente de correlação intraclasse; IC95%: intervalo de confiança de 95%.

The mean of the scores and their standard deviation for the domains and total score of each of the questionnaires are shown in Table 2. Pearson’s correlation coefficient used in the analysis of convergent and divergent validities between the QUALAS-C (Table 3) and QUALAS-T (Table 4) domains was low (r=0.35 and r=0.46, respectively), indicating that the domains of the instruments differentiate between two distinct components in the assessment of health-related QoL. Correlations between QUALAS-C and QUALAS-T domains and the KIDSCREEN-27 questionnaire were also low (r≤0.33 and r≤0.49), indicating that these instruments show different aspects of health-related QoL.

**Table 2:** Mean±standard deviation of the domains score and total score for QUALAS-C and QUALAS-T questionnaires.

Domain	QUALAS-C	QUALAS-T
Self-esteem and independence/Family and independence	79.2±18.2	77.8±22.3
Bladder and bowel	69.7±23.9	58.2±28.2
Total	149.0±34.8	136.0±43.3

**Table 3:** Pearson’s coefficient values between the QUALAS-C domains and the KIDSCREEN-27 scores.

	Domain	QUALAS-C		KIDSCREEN-27				
		Self-esteem and independence	Bladder and bowel	Physical health	Psychological health	Autonomy and family	Friends	School and learning
QUALAS-C	Self-esteem and independence	1.00	-	-	-	-	-	-
	Bladder and bowel	0.35^a^	1.00	-	-	-	-	-
KIDSCREEN-27	Physical health	-0.10^c^	0.11^b^	1.00	-	-	-	-
	Psychological health	0.30^b^	-0.05^b^	0.08^b^	1.00	-	-	-
	Autonomy and family	0.19^b^	0.18^b^	-0.02^b^	-0.01^b^	1.00	-	-
	Friends	0.16^b^	0.17^b^	0.22^b^	0.22^b^	0.19^b^	1.00	
	School and learning	0.33^a^	0.25^b^	0.04^b^	0.26^b^	0.20^b^	0.10^b^	1.00

^a^p≤0.05; ^b^p≥0.10; ^c^0.05<p<0.10.

**Table 4:** Pearson’s coefficient values between the QUALAS-T domains and the KIDSCREEN-27 scores.

	Domain	QUALAS-T		KIDSCREEN-27				
		Family and independence	Bladder and bowel	Physical health	Psychological health	Autonomy and family	Friends	School and learning
QUALAS-T	Family and independence	1.00	-	-	-	-	-	-
	Bladder and bowel	0.46^a^	1.00	-	-	-	-	-
KIDSCREEN-27	Physical health	0.37^a^	0.32^a^	1.00	-	-	-	-
	Psychological health	-0.06^b^	0.01^b^	0.26^b^	1.00	-	-	-
	Autonomy and family	0.16^b^	0.15^b^	0.23^b^	0.16^b^	1.00	-	-
	Friends	0.22^b^	0.34^a^	0.30^c^	0.38^a^	0.36^a^	1.00	
	School and learning	0.41^a^	0.49^a^	0.27^c^	0.42^a^	0.26^b^	0.54^a^	1.00

^a^p≤0.05; ^b^p≥0.10; ^c^0.05<p<0.10.

## DISCUSSION

This study aimed to translate, adapt, and evaluate the psychometric properties of reliability and validity of the Brazilian version of the QUALAS called “*Avaliação da Qualidade de Vida em Espinha Bífida*” in its versions for children aged 8 to 12 (QUALAS-C) and for adolescents aged 13 to 17 (QUALAS-T).^5^ ^,^ ^6^ Compared to other specific health-related QoL instruments in patients with spina bifida, the QUALAS questionnaire is the first to present a self-applicable version for children under 13 years of age. It also addresses the issue of urinary and fecal incontinence, which are important aspects for the QoL of this specific population.

The questionnaire adaptation by the commission was characterized as an important step to identify terms that could not be understood by the target population, allowing them to be altered before being back-translated.^9^ The translation of the questionnaires needed some modifications after its analysis by the commission. This fact reinforces the necessity of the translation and cultural adaptation stages of questionnaires for the Brazilian population in a way that facilitates the understanding of patients, increasing the possible applicability of an instrument, even though the modifications made in the questionnaire were considered small.^10^

The QUALAS questionnaire for both populations showed easy cultural adaptation and good reliability (internal consistency and reproducibility). The validity of the convergent and discriminant construct was adequate in both the questionnaire for children (QUALAS-C) and the other for adolescents (QUALAS-T).

The internal consistency of the QUALAS-C questionnaire assessed by the Cronbach’s alpha coefficient calculation was adequate for the total score (alpha=0.73). When we evaluated the internal consistency per domain, we found a below-adequate value for the Self-esteem and independence domain (alpha=0.56) and adequate for the Bladder and bowel domain (alpha=0.74). A low coefficient may mean that the domain items are not suitable for the construct to be evaluated, in this case the QoL.^11^ In the original article, the internal consistency values for each domain were 0.72 and 0.74, respectively.^5^

The QUALAS-T questionnaire presented adequate internal consistency for both the total score (0.79) and its two domains (Family and independence = 0.71, Bladder and bowel = 0.74). The internal consistency values of the domains were similar to those found in the original QUALAS-T questionnaire validation article (0.76 and 0.78, respectively).^6^

The present study demonstrates high stability in the reproducibility of both questionnaires (QUALAS-C - ICC=0.86 - and QUALAS-T - ICC=0.92), with higher values even in the evaluation of individual domains in relation to the original articles.^5^ ^,^ ^6^ Each evaluation instrument should reproduce over time the same result in two or more administrations to the same patient, considering that his/her clinical state has not changed.

The original and adapted instruments have not found similar values when compared to their psychometric properties, as well as in the relative weight of each item in the scale. This does not indicate that the translated version cannot be considered valid, but rather that an item does not carry the same cultural weight in both.^12^

The convergent and divergent validities that evaluate the concept of independence or redundancy were adequate in the QUALAS-C questionnaire, since the Pearson’s correlation coefficient values between its domains (r=0.35) and in relation to the domains of the KIDSCREEN-27 questionnaire (r<0.33) were low. A low correlation between the two questionnaires was expected to be found since the KIDSCREEN-27 instrument was developed to evaluate children in general, healthy or not, thus evaluating different aspects of health-related QoL.

In the analysis of the convergent and divergent validities of the QUALAS-T questionnaire, adequate values were also found, considering the Pearson’s correlation coefficients between its domains (r=0.46) and in relation to the domains of the KIDSCREEN-27 questionnaire (r<0.49) were low.

Even though it was not one of the study objectives, when comparing the QUALAS-C questionnaire scores with their original version per domain, we observed that in the original version the average score of the Self-esteem and independence domain was lower than in the current study (71.6 *versus* 79.2). On the other hand, in the Bladder and bowel domain, the mean score was slightly higher in the original study (70.7 *versus* 69.7).^5^ The mean scores for the QUALAS-T questionnaire per domain in validation when compared to the original instrument presented a different pattern to that observed in the QUALAS-C questionnaire: mean score of the Family and independence domain was higher than in the original study (77.8 *versus* 57.0) and mean score of the Bladder and Bowel domain was lower compared to the original study (58.2 *versus* 68.5).

In conclusion, after performing the stages of translation, cultural adaptation, and evaluation of the psychometric properties of the QUALAS-C and QUALAS-T instruments, both versions of the QUALAS questionnaire proved to be valid and reliable and can be used to evaluate the health-related QoL of the Brazilian population of spina bifida patients aged between 8 and 17. We acknowledge as a potential limitation the fact that patients were recruited from the same region/area and may not represent the overall cohort of patients with myelomeningocele in the country.
